# A Redox-Probe-Free Immunosensor Based on Electrocatalytic Prussian Blue Nanostructured Film One-Step-Prepared for Zika Virus Diagnosis

**DOI:** 10.3390/bios12080623

**Published:** 2022-08-10

**Authors:** Lorenna K. B. Santos, Priscila D. Mendonça, LiLian K. S. Assis, Carlos R. Prudêncio, Maria Izabel F. Guedes, Ernesto T. A. Marques, Rosa Fireman Dutra

**Affiliations:** 1 Biomedical Engineering Laboratory, Department of Biomedical Engineering, Federal University of Pernambuco, Avenida Professor Moraes Rego 1235, Recife 50670-90, Brazil; 2 Immunology Center, Institute Adolfo Lutz, São Paulo 01246-902, Brazil; 3 Human Biochemistry Laboratory, University of Ceará, Fortaleza 60714-903, Brazil; 4 Department of Infectious Diseases and Microbiology, University of Pittsburgh, Pittsburgh, PA 15261, USA

**Keywords:** Zika virus, immunosensor, Prussian blue, envelope protein, point-of-care diagnosis

## Abstract

The Zika virus (ZIKV) is a great concern for global health due to its high transmission, including disseminating through blood, saliva, urine, semen and vertical transmission. In some cases, ZIKV has been associated with microcephaly, neurological disorders, and Guillain–Barré syndrome. There is no vaccine, and controlling the disease is a challenge, especially with the co-circulation of the Dengue virus, which causes a severe cross-reaction due to the similarity between the two arboviruses. Considering that electrochemical immunosensors are well-established, sensitive, and practical tools for diagnosis, in this study we developed a sensor platform with intrinsic redox activity that facilitates measurement readouts. Prussian blue (PB) has a great ability to form electrocatalytic surfaces, dispensing redox probe solutions in voltammetric measurements. Herein, PB was incorporated into a chitosan–carbon nanotube hybrid, forming a nanocomposite that was drop-casted on a screen-printed electrode (SPE). The immunosensor detected the envelope protein of ZIKV in a linear range of 0.25 to 1.75 µg/mL (*n* = 8, *p* < 0.01), with a 0.20 µg/mL limit of detection. The developed immunosensor represents a new method for electrochemical measurements without additional redox probe solutions, and it is feasible for application in point-of-care diagnosis.

## 1. Introduction

The Zika virus (ZIKV) is considered endemic and an emerging disease of great concern for health services, due to its high transmission rates in the tropical regions of Africa, America, and Asia [1]. In addition to vector transmission by mosquitoes of the *Aedes* genus and other arthropods, ZIKV can be transmitted by sexual intercourse, blood transfusion, saliva, urine, and vertical transmission, which makes controlling its spread more difficult [2]. ZIKV infection is a self-limiting disease; although in most cases it ranges from being asymptomatic to presenting mild symptoms such as fever, joint pain, headache, malaise, and rashes, it can also develop into a severe form [3]. In 2016, during a large outbreak in America, especially in northeastern Brazil, the correlation between the potential mechanism of severe congenital microcephaly and malformations in infants was confirmed [4,5]. Additionally, records of spontaneous abortions and decreased male fertility have also been associated with ZIKV infection [6,7].

Currently, the gold standard for ZIKV diagnosis is the molecular method, whereby the viral RNA is detected using the reverse transcription technique (PCR) [8]. However, molecular tests have seen limited use in several endemic countries due to the high cost of analysis and require skilled laboratory staff for sample preparation and result managements [9]. Furthermore, molecular tests are suitable only in the acute stage, when the replication phase of the virion is still highly active [1]. After the viremic period, serological assays to detect antibodies or virion proteins of ZIKV are more appropriate. ZIKV is a member of the *Flaviviridae* family, with a positive-sense RNA genome codifying seven non-structural proteins (NS1, NS2A, NS2B, NS3, NS4A, NS4B, and NS5) and three structural proteins, which are cleaved into the capsid (C), envelope (E), and precursor membrane (prM) [10]. The serological detection of ZIKV can identify any of these proteins or antibodies from the humoral response. However, in many countries, multiple viruses are in co-circulation, including Dengue, Zika, Yellow Fever, and West Nile. It is challenging to eliminate the cross-reactivity among these viruses due to their similarities, especially to distinguish Dengue and Zika [11,12]. Recently, the ZIKV E protein has been identified as a good biomarker for ZIKV since it is the largest of the virion and is involved in many aspects of the viral cycle, including membrane fusion and binding mediation, which makes it an optimal choice for serological diagnosis [1]. The ZIKV E protein stands out among the structural proteins, as it offers greater power to differentiate between ZIKV and other flaviviruses [13].

Immunochromatographic tests (ICTs) have been identified as an attractive tool for rapid diagnosis, especially during outbreaks, as was evidenced recently in the Coronavirus pandemic beginning in March 2020 [14]. ICTs have the advantage of producing results quickly (within approximately 15–30 min), and most can be used as a point-of care test with greater usability ICTs have become popular and are distributed in many countries, facilitating rapid disease management and intervention and helping countries with limited or poor resources [15,16]. Nowadays, several ZIKV tests using chromatography technology are commercially available, such as the Chembio DPP Zika IgM system (DPP Zika ICA; Chembio, Medford, NY, USA) and others that detect antigens or antibodies in blood and saliva [17]. Although ICTs have many advantages, they are limited in terms of sensitivity compared to laboratory immunoassays that perform detection using enzymatic reactions (ELISA) or electrochemiluminescence (ECLIA); in addition, these analyzers can provide quantitative results, and their sensitivity and specificity are higher than those of ICT-based devices. However, they also have several disadvantages, such as high infrastructure costs, additional steps for sample processing, the longer time required to obtain results, and the need for sophisticated equipment that substantially increases the cost per test, restricting their use in ZIKV-endemic areas and their distribution in places with limited resources. In contrast, immunosensors based on nanomaterials can mitigate all these drawbacks [18] while achieving the same sensitivity as ELISA immunoanalyzers, and they have the advantage of being a portable technology, thus facilitating therapeutic management.

In recent years, several immunosensors for ZIKV have been developed with optical [19,20] and acoustic mechanisms, using multimode interference waveguides [21] and electrochemical transduction [17,22]. Although notable advances in immunosensor technologies have been observed, certain challenges and drawbacks still need to be overcome for their application in clinical practice, such as the many steps required and their unsuitability for practical use. In this study, we designed a redox-free electrochemical sensor by exploring Prussian blue (PB) as a stable electroactive species intrinsically immobilized on the sensor surface. Species derived from the oxidation of PB are capable of generating a faradaic current response, creating a diffusional charge flow at the sensor surface. Based on our previous studies and those of other authors using active redox surfaces [22,23,24], this new design provides faster responses, a simpler readout system, and fewer steps to avoid passivation on the sensor surface and minimize false responses. PB is a chemically recognized compound that promotes redox reactions in a reversible way [25]. It has been denoted as an “artificial peroxidase”, since it is a chemical with high catalytic activity. However, if PB is simply adsorbed onto the sensor surface, a loss of reversibility can easily occur, as this chemical is sensitive to cyclic potential variations or even simple changes in pH [26]. One strategy is its incorporation into compatible polymers that allow anchoring and stability.

Chitosan (CHI) is a cationic polysaccharide rich in amine groups that allow the stable immobilization of proteins by amide bonds [27,28]. Herein, CHI was used to disperse carboxylated carbon nanotubes (CNT) to form a nanocomposite, CHI–CNTs@PB. The nanostructured film on the SPE was obtained by drop-casting in a single step, facilitating scale-up. Different concentrations of PB were evaluated to obtain a thin film with good conductivity. The ZIKV E protein was detected by the reduction of the faradaic current without an additional redox probe solution in the electrochemical procedure, such as the well-known potassium ferri/ferrocyanide.

## 2. Materials and Methods

### 2.1. Reagents and Materials

Iron (III)-ferrocyanide-Fe4 (Fe (CN) 6) 3 (PB), chitosan (CHI), glycine, potassium ferricyanide (K3 (Fe [CN] 6)), and potassium ferrocyanide (K4 (Fe [CN] 6)) were obtained from Sigma-Aldrich (St. Louis, MO, USA). Recombinant ZIKV E protein and anti-ZIKV E protein antibodies were acquired from EastCoasr Bio (North Berwick, UK). The ZIKV Vero E6 tissue culture antigen was acquired from Bei Resources (Manassas, VA, USA). Multi-walled carbon nanotubes (CNTs) and screen-printed electrodes (SPEs) were obtained from Dropsens (Oviedo, Spain). Tris Hydrochloride buffer (Tris-HCl) (100 mmol·L^−1^, pH 6.5) was used in the blocking solution containing glycine (100 mmol·L^−1^). Phosphate buffer saline (PBS) (100 mmol·L^−1^, pH 7.4) was used in all experiments to dilute the samples. All other chemicals used were of analytical grade, and solutions were prepared in ultra-pure water acquired from a MilliQ station (USA) and had resistivity below 18 MΩ cm^−1^.

### 2.2. Native and Recombinant ZIKV Antigen Spiked Serum Samples

A serum pool was prepared by mixing seven serum samples obtained from volunteers collected by venipuncture. Blood samples (4 mL) were collected in a dry tube (vacutainer) and subjected to centrifugation at 2000× *g* for 20 min to obtain the serum. All serum samples were freshly processed within 2 h of collection. Lipemic and hemolyzed sera were discarded to avoid false responses. Serum samples were previously confirmed to be negative for ZIKV by molecular diagnostics RT-PCR and IgM ELISA following the assay protocol of the US Centers for Disease Control and Prevention (CDC). All participating volunteers were duly informed, and the protocols were approved by the CPqAM/FIOCRUZ Ethics Committee (protocol number 63441516.6.0000.5190).

To evaluate the analytical responses, the electrode was subjected to the ZIKV E recombinant protein as well as the native antigen extracted from the viral culture. First, antigens were diluted in PBS (100 mmol·L^−1^, pH 7.4), and afterward, the pools of serum were 1:4 diluted in PBS that had previously been spiked with the respective analytes. Control samples (unspiked pool of serum) were obtained under the same conditions, except Vero-E6 cells without the virus were used.

### 2.3. Electrochemical Measurements

Electrochemical measurements were performed in the Autolab potentiostat/galvanostat model PGSTAT204 from Eco Chemie (Utrecht, The Netherlands), interfaced to the PC system and controlled by Nova 2.1.1 software. Carbon ink SPEs (DRP-C110) were purchased from Dropsens (Spain). The diameter of the working electrode was 4 mm, allowing micro-volume samples. The measurements were performed at room temperature (~24 °C) by pipetting 25 μL of the sample volume on the screen-printed electrode, which was interfaced to the potentiostat by a DSC cable connector (Dropsens, Spain).

Electrochemical characterizations of the surface electrode modifications were performed by cyclic voltammetry (CV) in a window potential between −0.4 and 0.6 V with a scan rate of 50 mV·s^−1^, applying a pre-treatment of 5 s at −0.3 V potential to stabilize the residual current. Analytical responses were also evaluated by CV under the same conditions and measured by the anodic current peaks with blank subtraction (before sample incubations). Characterizations and analytical responses, unless stated otherwise, were measured in the presence of KCl (100 mmol·L^−1^) as the supporting electrolyte. All statistical analyses and graphs were processed using OriginPro version 9.0 software (OriginLab Co., Northampton, MA, USA).

Before chemical modifications for antibody immobilization, screen-printed electrodes (SPE) were cleaned in ethanol and ultra-pure water. After cleaning, they were electrochemically pre-treated using a cyclic voltammetry procedure, in which the SPEs were subjected to a potential window between −1.8 and 1.8 V at a scan rate of 80 mV·s^−1^ in the presence of 100 mmol·L^−1^ KCl solution.

### 2.4. Structural and Morphological Analyses

For morphological characterizations, micrographs were obtained by scanning electron microscopy (SEM) using an AURIGA model with crossbeam scanning (Zeiss, Jena, Germany). The microscope contained a field emission gun (FEG) electron column and a focused ion column (FIB) that provided better image quality compared to the traditional filament sources. MEV was operated at a potential of 0.1–30 kV and a resolution of 1 nm at 15 KV. Samples were deposited onto stubs and secured with glassy carbon tape. Before obtaining the images, the stubs containing the samples were metallized for a period of 90 s using a sputtering coater (model Q15OR PLUS, Quorum Technologies LTD, Lewes, UK). The resolutions of 200 nm and 1 µm were chosen to obtain the images.

### 2.5. Synthesis of the CHI–CNT@PB Nanocomposite

The preparation of the CHI–CNT@PB nanocomposite solution was carried out through the mixture of solutions A and B. Solution A was prepared by adding 3 mg CHI to 3 mg CNTs, dissolved in 2 mL acetic acid (2.5% *w*/*v*), and homogenizing in an ultrasound bath for 1 h at room temperature (~25 °C). Solution B was obtained by dispersing 12 mg PB in 1 mL of 2.5% (*v*/*v*) acetic acid and subjecting the mixture to an ultrasound bath for 1 h at room temperature (~25 °C). The final mixture was obtained by adding solution A to solution B, resulting in a total volume of 3 mL. This mixture was subjected to an ultrasound bath for 1 h at room temperature (~25 °C), thus forming the CHI–CNT@PB nanocomposite with final concentrations of 1 mg·mL^−1^ CHI, 1 mg·mL^−1^ CNTs, and 4 mg·mL^−1^ PB. 

### 2.6. Assembly of the CHI–CNT@PB Nanoelectrode SPE

**Figure 1:**
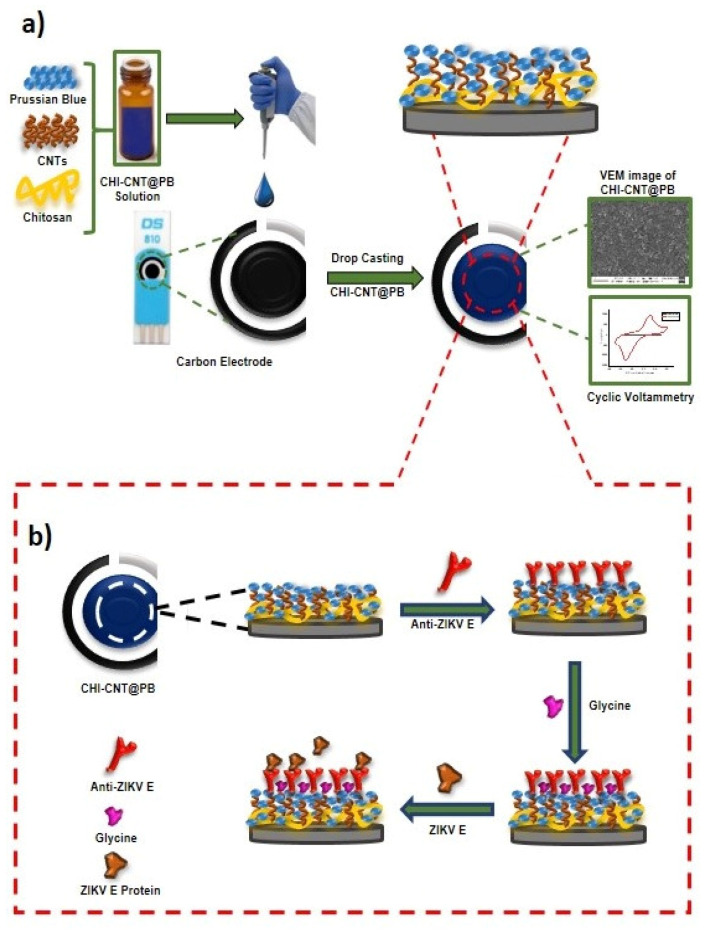
Diagram of the ZIKV immunosensor: (**a**) steps performed in the synthesis of the nanostructured electrode and (**b**) process of immobilization of the anti-ZIKV E protein and immunoassay.

Before assembling the CHI–CNT@PB nanocomposite film, SPEs were cleaned using a cyclic voltammetry procedure, in which the SPEs were subjected to a potential window between −2.0 and 2.0 V at an 80 mV·s^−1^ scan rate in the presence of the 100 mmol·L^−1^ KCl solution; this was followed by rinsing with ultra-pure water and air-drying at room temperature (~24 °C). Then, 7.5 µL of the prepared CHI–CNT@PB nanocomposite solution was pipetted onto the SPE working surface, and the modified SPE was subjected to oven-drying at 50 °C for 10 min. Four layers were drop-casted using a total volume of 10 µL nanocomposite per electrode (Figure 1a). 

### 2.7. Immobilization of Anti-ZIKV E and Immunoassay

First, an aliquot of 5 µL of anti-ZIKV E protein solution at 0.5 µg·mL^−1^ concentration prepared in PBS (pH 7.4) was deposited on the working SPE surface and incubated in a moist chamber for 1 h at 60 °C. Afterward, a blocking solution, formed from glycine (100 mmol·L^−1^) prepared in Tris-HCl (pH 6.5), was pipetted onto the working SPE surface and incubated for 2 h. Finally, the immunosensor was washed in PBS at 100 mmol·L^−1^ and stored in a refrigerator (~8 °C) until use.

Analytical responses were obtained by incubating the Env ZIKV (0.25 µg·mL^−1^) on the surface of the SPE for 20 min for each measurement. Before measurements, the electrodes were immersed for 10 s in a beaker containing 10 mL of PBS with medium agitation. The immunosensor preparation step is illustrated in detail in Figure 1b.

## 3. Results and Discussion

### 3.1. CHI–CNT@PB Nanocomposite

#### 3.1.1. Synthesis and Optimization of Experimental Conditions

PB has been widely used in the development of electrochemical biosensors due to its catalytic activity. However, in most cases, PB and its derivatives are unstable when subjected to cyclic voltammetry, potential variations, and pH changes—parameters that compromise the diagnostic sensitivity and specificity of biosensors. The search for alternatives to improve PB stability without damaging its electrocatalytic properties has been previously described [24,29]. In this study, a CHI polymer was used due to its well-known biocompatibility and ability to immobilize biomolecules with amine groups. Additionally, CHI can confer increased stability to films aggregating on anionic surfaces and thus can easily bind to PB [30]. Although CHI is considered a non-conductive polymer, it can improve electron transfer when incorporated into materials with good electrical conductivity; however, its use in the construction of nanocomposites must be considered carefully so that it provides stability without a significant decrease in the electrical signal [31,32,33].

**Figure 2:**
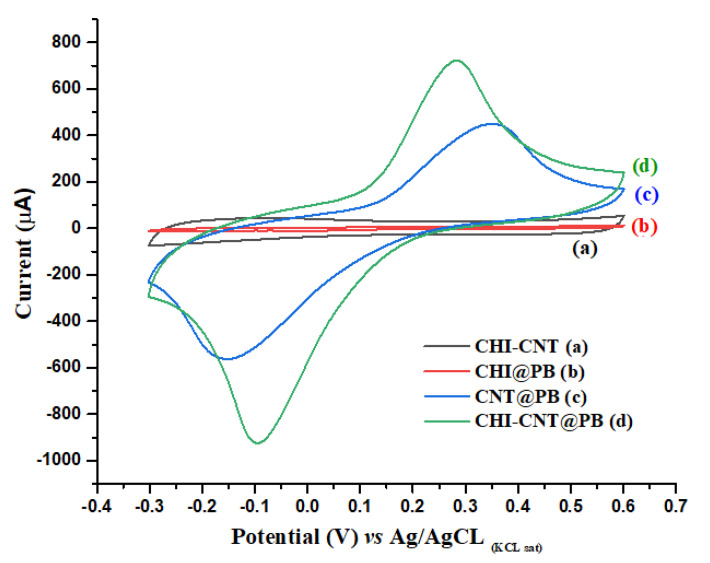
Cyclic voltammograms at 0.1 V·s^−1^ scan rate with different film compositions: (**a**) CHI–CNT, (**b**) CHI–PB, (**c**) CNT–PB, and (**d**) CHI–CNT@PB. Measurements were performed in the presence of KCl (100 mmol·L^−1^) at a 0.05 V·s^−1^ scan rate.

Cyclic voltammetry (CV) is an electrochemical method widely applied in studies for understanding electron transfer processes. In this study, the electrocatalytic activity of the CHI–CNT@PB film component was verified by the presence of redox peaks in measurements with 100 mmol·L^−1^ KCl as an electrolyte support (Figure 2). The electrocatalytic activity of PB in the CHI–CNT@PB film was evaluated by comparing it with the following control experiments: (a) CHI + CNT, (b) CHI + PB, (c) CNT + PB, and (d) CHI–CNT@PB. The redox activity of PB was confirmed in curves (c) and (d) of Figure 2 by the presence of redox peaks. Although PB was present in curve (b), redox peaks were not observed. This was attributed to the CHI + PB being synthesized in a gel with insulating properties, since it was not evidenced in the PB current peaks [34]. The formation of anodic and cathodic peaks was observed in curve (c) at the potentials of 0.36 V and −0.15 V, respectively, which was attributed to the redox processes resulting from the CNT–PB interactions with a remarkable electrocatalytic effect [35]. There was also a great increase in the electroactive area; however, the composite drop-casted on the electrode surface showed instability when subjected to successive CVs, suggesting that the polymers promoted greater stability in the nanocomposite.

Films containing CNTs produced by polymers have shown good stability in the construction of biosensor platforms [36]. Here, CNT was incorporated with CHI, by using a CNT–CHI/PB film, which demonstrated anodic and cathodic peaks at the 0.3 V and −0.1 V potentials, respectively (curve d), representing an increase of approximately 53 % in the electroactive surface area (cm^2^) compared to CNT–PB without CHI. This increase in the electroactive area of the CNT–CHI/PB was determined by CVs according to the Randles–Sevcik equation:

Ip = (2.687 × 10^5^) n ^(3/2)^ A·C_o_ (D·ν) ^(1/2)^ (1)

where Ip is the current peak, n is the number of electrons transferred, A is the electrode area (cm^2^), C_o_ is the molar concentration of the redox species (mol·cm^−3^), D is the diffusion coefficient of PB, and ν is the scan rate (V·s^−1^). According to Equation (1), the current peak is directly proportional to the concentration, and its increase is related to the scan rate [37,38]. Conversely, the CNT–PB film without the presence of CHI did not produce peaks. It is believed that this significant increase in the electroactive area was due to the CHI–CNT@PB synthesis process, in which the PB catalytic sites were more exposed to the nanocomposite surface compared to the mixture of all components simultaneously. In this case, the CNTs were first dispersed in the CHI solution, and afterward, the PB was added. This conductivity increase was also attributed to the strong interaction between the cationic groups of the CHI and the PB, which present opposite charges, resulting in a synergistic effect. The cationic groups of the CHI, when undergoing oxidation–reduction at their respective potentials, transfer ionic species that help restore the redox processes of the BP, guaranteeing its good reversibility. This was confirmed by the redox peaks in the CVs of the CHI–CNT@PB film at −0.1 and 0.27 V for the cathodic and anodic potentials, respectively (Figure S1). The stability of the electrocatalytic nanocomposite on the electrode surface was evaluated by subjecting the modified electrode to 30 successive CVs. The coefficient of variation of the anodic and cathodic current peak amplitudes were 2% and 3%, respectively, indicating a good performance (CV < 5%).

**Figure 3:**
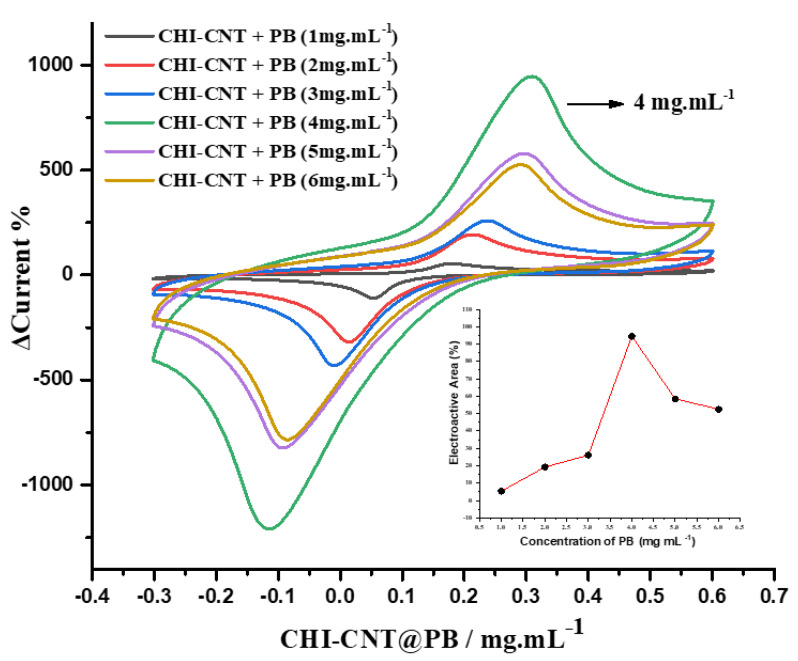
CV profiles according to the PB concentration (inset: electroactive area as function of different PB concentrations). Measurements performed in 100 mmol·L^−1^ KCl at 0.05 V·s^−1^ scan rate.

**Figure 4:**
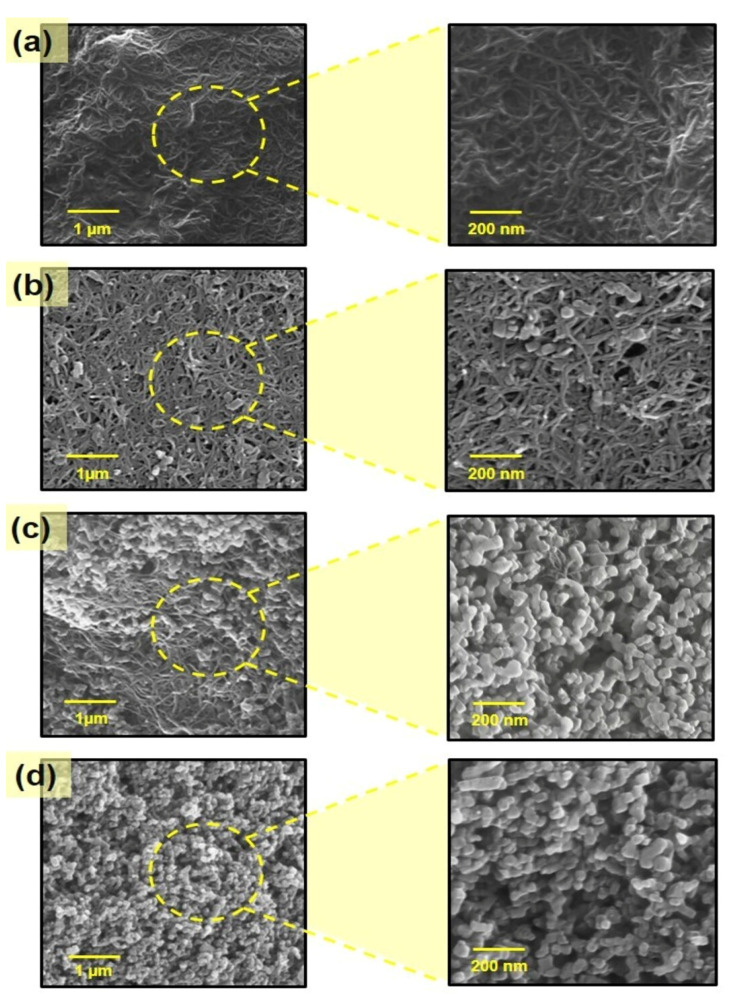
SEM micrographs of the following: (**a**) CHI–CNT, (**b**) CHI–CNT@PB, (**c**) CNT–PB, (**d**) CHI–PB.

A study of the optimal concentration of PB for generating faradaic currents was conducted (Figure 3). Significant growth was observed in the redox peak pairs with an increase in the concentration of PB, reaching the maximum value at the concentration of 4 mg·mL^−1^ (inset Figure 4). This increase was attributed to the synergistic effect between the CHI–CNT–PB components, since the carboxylic groups present in CNTs are electrostatically attracted by the amine groups present in CHI that are bound to the PB. The investigation of the optimal PB concentration in the nanocomposite showed that at a concentration above 4 mg·mL^−1^, the peak currents were reduced; this suggested that higher concentrations exceeded the number of available binding sites, causing excess PB to detach from the film, thus reducing the electrocatalytic activity and stability. These materials were dispersed in an acidic medium, since PB contains ions that are more easily hydrolyzed in a neutral medium, to stabilize the chemical bonds between the compounds [39].

#### 3.1.2. Morphological Characterization

**Figure 5:**
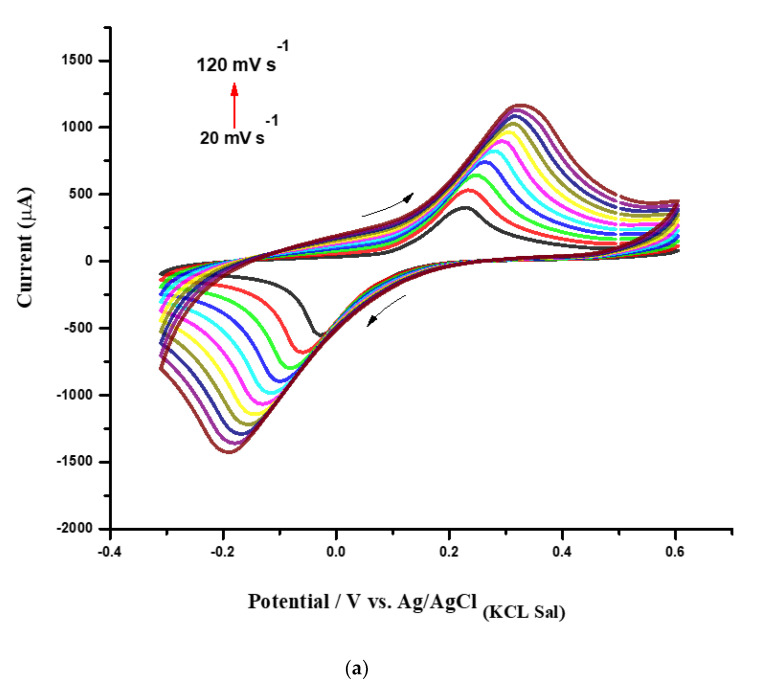

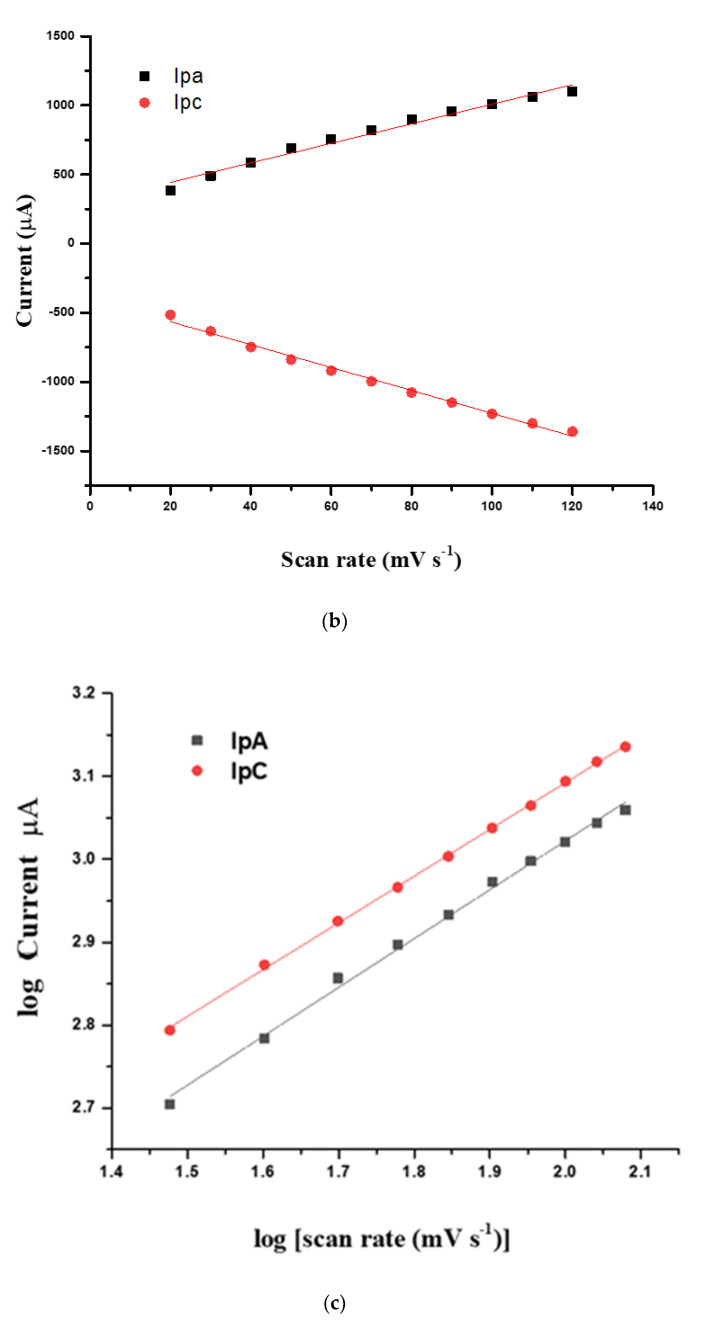
(**a**) CVs obtained from the CHI–CNT@PB film at 20, 30, 40, 50, 60, 70, 80, 90, 100, 110, and 120 mV·s^−1^ scan rates (inner to outer); (**b**) plot of anodic and cathodic peak currents from CVs vs. square root of scan rate; (**c**) plot of log Ipa and Ipc vs. log scan rate. All measurements were performed in KCl (100 mmol·L^−1^) as the supporting electrolyte.

Scanning electron micrography was performed to study the conformational structures of the CNT–PB, CHI–CNT, CHI–PB, and CHI–CNT@PB films (Figure 4). PB is a porous and inorganic material formed by iron and hexacyanoferrate bridges. It demonstrates high redox activity, reversible electron transfer behavior, and excellent electrocatalytic activity. These characteristics make its use convenient and promising for the development of sensor platforms [29]. Figure 4a shows the morphology of the CHI–CNT film, formed of tubular structures compatible with the CNTs covered by the CHI polymer, which appeared on a cohesive and dense base. Yu et al. (2020) reported that the binding of CNTs to CHI occurs through adsorption by the carboxyl groups of the CNTs to the amine groups of the CHI. Although a uniform nanocomposite was observed, forming a homogeneous film with a greater surface area at the expense of the nanomaterial, the insulating nature of the CHI was predominant and therefore reduced the electrocatalytic activity [40]. To promote electrocatalytic activity by the adsorption of a redox species to the film, PB was added to the CHI–CNT film, and the synthesis was carried out in two ways, either by mixing the three compounds simultaneously or by adding the PB after the dispersion of the compounds. CNTs were added to the CHI, and the mixture was subjected to an ultrasound bath. In the latter synthesis method, a greater increase in redox peaks was observed in the CVs, probably due to the greater exposure of the PB, which was bound to the cationic polymer of the CHI by the anions of hexacyanoferrate (II). Figure 4b shows the CHI–CNT@PB film, demonstrating that the PB was more superficially and clearly immobilized on the CHI–CNT film than on the other structures. BP is characterized by cluster structures of a cubic nature, as shown in detail in Figure 5b. As illustrated in Figure 4c, when CHI was not present, PB seemed to dissociate from the CNTs, presenting heterogeneous complexes of isolated structures. Such dissociation corroborates the findings of the CVs, which showed the electrocatalytic activity with prominent redox peaks [41]; however, this film did not demonstrate electrochemical stability when subjected to the same conditions and number of cycles as the CHI–CNT@PB films. According to Figure 4d, when analyzing the CHI–PB composite, the film morphology showed an aggregated appearance with small particles. This porosity was due to the interaction between the cationic chains of the chitosan and the Fe anionic ions of the PB.

#### 3.1.3. Study of Electrochemical Kinetic Properties

Figure 5a presents the CV profiles corresponding to the modified SPE with the CHI–CNT@PB nanocomposite submitted to different scan rates, ranging from 20 mV·s^−1^ to 120 mV·s^−1^ at a potential window between −0.3 V and 0.6 V. These measurements were performed in 100 mmol·L^−1^ KCl, and the observed redox current peaks indicated the reversible activity of the PB (Figure 5a). It was noted that the anodic (Ipa) and cathodic current peaks (Ipc) increased according to the scan rate, forming a linear curve until approximately 120 mV·s^−1^. Correlation coefficients were calculated for Ipa (r = 0.988, *n* = 11) and Ipc (r = 0.996, *n* = 11), confirming that the electroactive PB was successfully surface-confined at the electrode (Figure 5b).

As shown in Figure 5a, a slight displacement in the redox peak potentials was observed as the scan rate increased, indicating that PB species were adsorbed into the film. Through a more accurate analysis, after plotting the logarithm of the scan rate versus the logarithm of the cathodic and anodic peak currents, it was observed that the slopes of the regression linear curves were 0.56 and 0.54, respectively (Figure 5c). This meant that the confined species generated on the electrode interface by redox reactions with the catalytic PB resulted in a diffusion-controlled process, since the obtained values were very close to the theoretical value of 0.5, which represents an ideal reaction of the diffusion-controlled electrode process [23].

### 3.2. Assembly of the Anti-ZIKV E Immunosensor Platform

**Figure 6:**
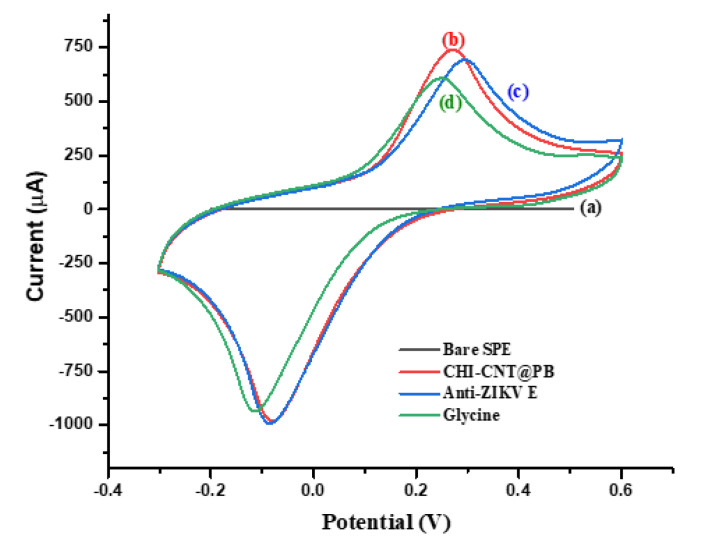
CVs of the stepwise preparation of the immunosensor: (**a**) bare SPE, (**b**) CHI–CNT@PB, (**c**) Anti-ZIKV E, (**d**) glycine (100 mmol·L^−1^). Measurements were performed in the presence of KCl (100 mmol·L^−1^) for a 0.05 V·s^−1^ scan rate.

The assembly of the biosensor platform was followed by the measurement of the voltammetric profiles without a redox solution in the presence of the supporting electrolyte of 10 mmol·L^−1^ KCl, including an analysis of the anodic peak currents and electroactive area (Figure 6). The electrode modified with the CHI–CNT@PB film showed an electrocatalytic profile with dramatic increases in the current redox peaks (curve b), especially compared to the cleaned electrode, in which the currents were practically zero (curve a). The immobilization of anti-Env ZIKV antibodies was confirmed by the reduction in the anodic peaks due to the insulating nature of the proteins (curve c) [42]. Given the cationic characteristics of CHI, anti-Env ZIKV antibodies were probably immobilized in an oriented mode, e.g., by their FC portion [38]. To block the nonspecific bindings, one aliquot (2 μL^−1^) of 100 molL^−1^ glycine solution was prepared in 100 mmol·L^−1^ Tris-HCl buffer (pH 6.5), pipetted onto the sensor surface, and left to react for 2 h. This step was important to avoid nonspecific bindings, which were confirmed by a decrease in the current redox peaks (curve d).

### 3.3. Analytical Responses to the ZIKV E Protein and ZIKV Isolated Culture

Various electrochemical immunosensors for ZIKV have been described, relying on the detection of the NS1 protein [43], NS2B protein [22], and membrane protein [44]. To the best of our knowledge, this work is the first to focus on the detection of the ZIKV envelope protein. Some immunosensors have a lower limit of detection, with the disadvantage of requiring a solution containing a redox probe for voltammetric measurements. Additionally, the E protein is more specific for ZIKV diagnosis than the NS1 protein, since NS1 has 50% homology with DENV [13]. NS2B is a redox-probe-free immunosensor developed by our group with attractive advantages in the acute stage of the disease [22].

**Figure 7:**
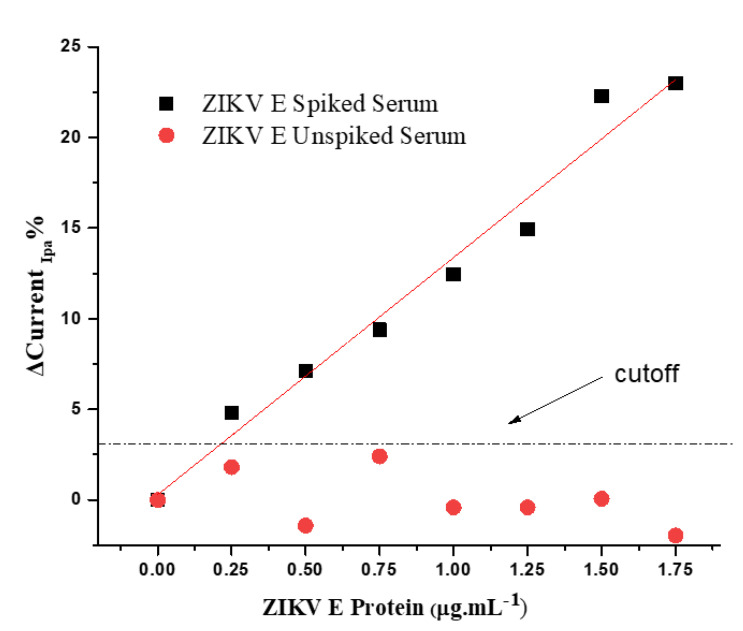
Analytical curve of the CV anodic peaks for immunosensor response to the serum pool spiked with ZIKV E protein diluted in PBS 100 mmol·L^−1^ (pH 7.4) and the unspiked serum pool diluted under the same conditions. Measurements were performed in the presence of KCl (100 mmol·L^−1^) for a 0.05 V·s^−1^ scan rate.

To evaluate the specificity of the immunosensor response, a control experiment was performed by incubating the electrode with both spiked and unspiked human serum samples. The pool was obtained from serum samples from seven healthy individuals, all free of lipemia and hemolysis, which can interfere with the immunosensor response. Under optimal conditions, the ZIKV immunosensor was subjected to successive incubations with spiked pool serum containing PBS-diluted ZIKV E protein, and CVs were registered (Figure S2). Current responses were measured according to the difference in the amplitude of the anodic peaks before and after incubation with ZIKV E protein in the pool serum. The analytical curve of the anodic current peaks was extracted from three ZIKV E protein standard curves (Figure 7); the values of the slope and intercept, expressed as the adjusted data for the linear equation, showed a Pearson correlation coefficient of approximately 0.98 (*p* < 0.01, *n* = 7, *t* test) with a low relative error (<1%). A linear range between 0.25 and 1.75 µg·mL^−1^ was obtained. The limit of detection was based on the least squares method, determined as three times the standard deviation of the current response intercept divided by the slope of the regression curve according to the IUPAC; the value obtained was 0.20 µg·mL^−1^ ZIKV E protein. This value falls in the clinical range for ZIKV diagnosis [19]. Unspiked ZIKV E serum was used as a control and tested under the same conditions, which resulted in a current response that was practically constant, indicating discrimination by anti-ZIKV protein E antibodies. The cutoff (limit of reaction) was obtained according to the average of the unspiked responses plus two standard deviations, resulting in a value of approximately 0.23 µg mL^−1^ ZIKV E protein. This experiment assessed the impact of interferences on the sensor response since the serum and pool samples contained different proteins, antibodies, and other components.

**Figure 8:**
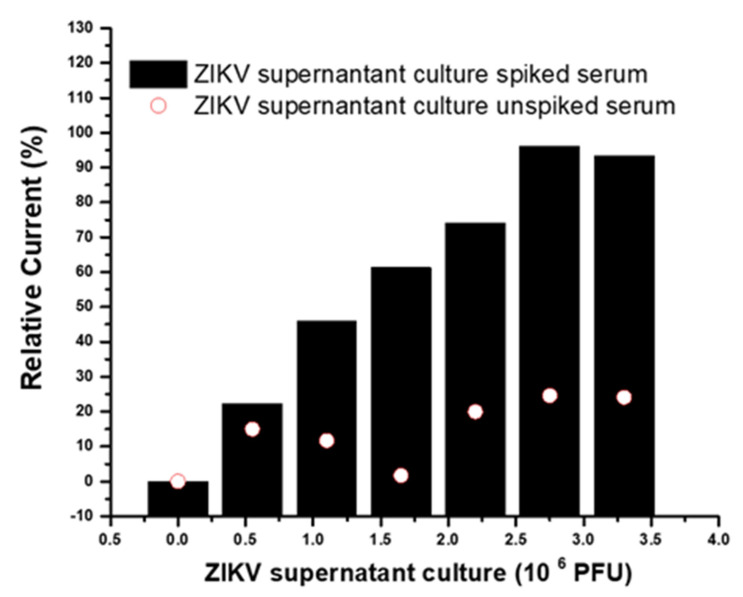
Immunosensor response to successive incubations with serum samples spiked and unspiked with ZIKV supernatant culture.

The evaluation response of the immunosensor to the virus culture, i.e., native ZIKV protein, was measured by subjecting the immunosensor to incubations with serum samples (pool) spiked with ZIKV cell culture supernatant (ZIKV Vero E6 Tissue Culture, Bioresource, Cotati, CA, USA). The pool was diluted 1:4 in PBS spiked with 0.55 × 10^6^ PFU. For analytical responses, the pool was successively incubated for 20 min on the electrode surface at room temperature (~24 °C). Before measurements, the electrodes were washed for 1 min in a PBS solution with stirring. Analytical responses were measured after successive incubations with spiked and unspiked samples and were compared (Figure 8). The linear range was from 0.55 to 2.75 × 10^6^ PFU, with a limit of detection of 0.18 PFU ZIKV. The analyses of the current responses showed statistically significant differences (*p* < 0.05) between spiked and unspiked samples after the application of the paired *t*-test, demonstrating that the immunosensor was specific and distinguished the ZIKV cell culture. Additionally, the analytical response of the immunosensor to the spiked sample was linear (coefficient correlation 0.979, *p* < 0.05), and the immunosensor showed a capacity to discriminate samples infected with the virus. The limit of the reaction (cutoff) for the native protein of ZIKV was less discriminative than that for the recombinant antigen. However, this was expected, since the antibody used was a monoclonal produced by the protein E recombinant antigen, and epitopes of the whole virus could be less exposed to the Fab portions of the anti-ZIKV E antibody. Thus, it is a promising immunosensor for application in real samples of infected patients.

### 3.4. Long-Term Stability

Immunosensors, over time, may undergo aging and the consequent loss of catalytic activity and decrease in sensitivity and specificity. Service life and stability studies are important to ensure the proper functioning and market success of these devices [3] The long-term immunosensor stability was evaluated over a period of 20 days using four readings from each electrode. Three electrodes were produced at the same time and stored in a moist chamber in a refrigerator at approximately 4–8 °C. For this study, four readings were taken on different days from each electrode (1st, 5th, 10th, and 20th days). For analytical responses, electrodes were subjected to incubations with a concentration of 0.25 μg mL^−1^ ZIKV-E-protein-spiked serum. The average relative standard deviations of the three electrodes were found to be 2.97%, which was considered promising for the immunosensor’s long-term stability.

## 4. Conclusions

An electrochemical immunosensor for detecting the Zika virus E protein was developed. The platform represents a new and effective method for detecting analytes without a redox probe, conferring the advantages of fewer steps and quicker analytical responses for immunosensors based on the analysis of faradaic currents. The redox-probe-free immunosensor was applied to the analysis of real samples of ZIKV-protein-E-spiked serum, demonstrating its ability to distinguish between positive and negative controls.

## Data Availability

Not applicable.

## Supplementary Materials

The following are available online at https://www.mdpi.com/article/10.3390/bios12080623/s1, Figure S1: Cyclic voltammograms of the CHI-CNT@PB nanocomposite film. The 30 cyclic voltammograms were performed in the presence of KCl (10 mmol·L^−1^) at a 0.05 V·s^−1^ scan rate; Figure S2: Anodic peaks of the CV by immunosensor response to the (a) Spiked pool of serum sample with ZIKV E protein and, (b) Unspiked pool of serum sample with ZIKV E protein.
