# Supplementary material for: A Redox-Probe-Free Immunosensor Based on Electrocatalytic Prussian Blue Nanostructured Film One-Step-Prepared for Zika Virus Diagnosis
Source: Biosensors (Basel). 2022 Aug 10;12(8):623. doi: 10.3390/bios12080623 (PMC9406047; doi:10.3390/bios12080623)
Supplement: Supplementary file 1 [file biosensors-12-00623-s001.zip › biosensors-1789744-supplementary.pdf]

Supplementary Information

A redox-probe free immunosensor based on electrocatalytic Prussian blue nanostructured film one-step prepared for Zika Virus diagnostic

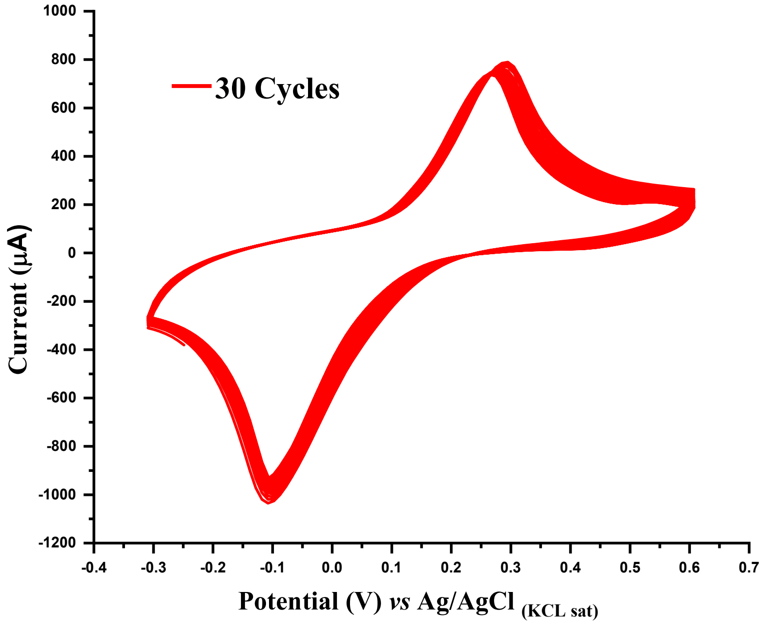

Figure S1. Cyclic voltammograms of the CHI-CNT@PB nanocomposite film. The 30 cyclic voltammograms were performed in the presence of KCl (10 $mmol.L^{-1}$) at a 0.05 V s^{-1} scan rate.

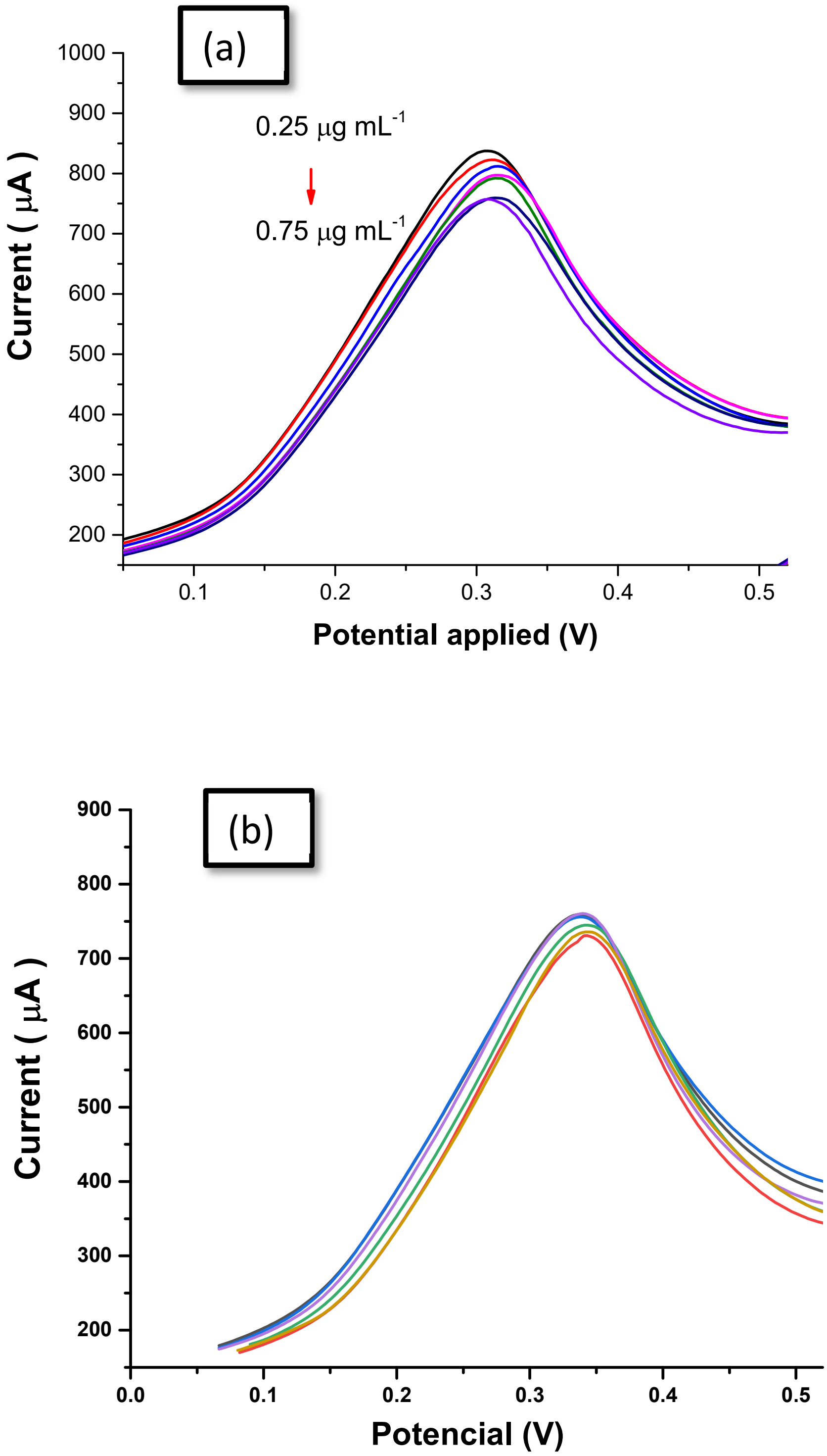

Figure S2. Anodic peaks of the CV by immunosensor response to the (**a**) Spiked pool of serum sample with ZIKV E protein and, (**b**) Unspiked pool of serum sample with ZIKV E protein.
